# Association Between Prognostic Nutritional Index and Clinical Outcomes After Percutaneous Coronary Intervention in Elderly Patients with Chronic Total Occlusion

**DOI:** 10.3390/jcdd13090464

**Published:** 2026-09-14

**Authors:** Jiquan Xiao, Song Wen, Renshang Xu, Qiheng Wan, Xiang He, Yusi Huang, Wenzhi Hu, Bin Zhang, Huimin Yu

**Affiliations:** 1 Department of Cardiology, Guangdong Cardiovascular Institute, Guangdong Provincial People’s Hospital, Guangdong Academy of Medical Sciences, Southern Medical University, Guangzhou 510080, China; 2 Department of Cardiology, Sun Yat-Sen Memorial Hospital of Sun Yat-Sen University, Guangzhou 510120, China; 3 Department of Cardiology, Fuwai Hospital, National Center for Cardiovascular Disease, Chinese Academy of Medical Sciences and Peking Union Medical College, Beijing 100037, China; 4 Department of Cardiology, Guangdong Provincial People’s Hospital’s Nanhai Hospital, Foshan 528000, China

**Keywords:** prognostic nutritional index, chronic total occlusion, percutaneous coronary intervention, all-cause mortality, major adverse cardiovascular events

## Abstract

Background: This study aimed to investigate the association between the prognostic nutritional index (PNI) and long-term all-cause mortality and major adverse cardiovascular events (MACEs) in elderly patients with chronic total occlusion (CTO) after successful percutaneous coronary intervention (PCI). Methods: This retrospective study enrolled 745 consecutive patients aged ≥ 60 years with successfully revascularized CTO between February 2011 and April 2023. All-cause mortality was the primary endpoint. MACE, defined as the first occurrence of all-cause mortality, non-fatal myocardial infarction, stroke, or target-vessel revascularization (TVR), was the secondary endpoint. Cox proportional hazards models and restricted cubic spline (RCS) analyses were used to evaluate associations. Results: During a median follow-up of 813 days, 58 (7.8%) all-cause mortality and 101 (13.6%) MACE occurred. In the primary multivariable model (Model 2), each 1-unit increase in PNI was associated with a 15% reduced risk of all-cause mortality (HR 0.85, 95% CI 0.81–0.90; *p* < 0.001) and an 8% reduced risk of MACE (HR 0.92, 95% CI 0.88–0.96; *p* < 0.001). Compared with the T1 group, patients in the T2 group had a significantly lower risk of all-cause mortality (HR = 0.21, 95% CI: 0.10–0.44; *p* < 0.001) and MACE (HR = 0.38, 95% CI: 0.23–0.63; *p* = 0.002). Similarly, the T3 group showed a significantly lower risk of all-cause mortality (HR = 0.24, 95% CI: 0.11–0.52; *p* = 0.003) and MACE (HR = 0.49, 95% CI: 0.30–0.82; *p* = 0.007). These associations remained directionally consistent in exploratory models with more extensive covariate adjustment. RCS indicated a linear association between PNI and all-cause mortality (*p* = 0.959) and a nonlinear association with MACE (*p* = 0.017). Conclusion: In this single-center retrospective cohort of elderly patients after successful CTO-PCI, lower baseline PNI was associated with higher risks of all-cause mortality and MACE. PNI may be a useful risk stratification tool, but its clinical utility requires confirmation in prospective, externally validated studies before implementation.

## 1. Introduction

Chronic total occlusion (CTO) lesions are common complex lesions in coronary angiography, defined as 100% occlusion of the lumen of the coronary arteries with thrombolysis in myocardial infarction (TIMI)grade 0 for forward flow lasting more than 3 months [1,2]. Percutaneous coronary intervention (PCI) for CTO is technically challenging; however, successful revascularization can effectively alleviate angina, improve cardiac function, and reduce long-term adverse events [3,4]. With the acceleration of population aging, the proportion of elderly CTO patients is increasing. Elderly patients often have complex conditions such as multiple cardiovascular risk factors, multiple vessel lesions, cardiorenal insufficiency, and malnutrition, and their long-term prognosis after PCI is worse than that of younger patients [3,5,6]. Therefore, identifying simple and reliable prognostic markers is crucial for risk stratification and individualized management of elderly CTO patients.

The prognostic nutritional index (PNI), calculated from serum albumin and lymphocyte count, was originally developed as an immunonutritional index [7]. In cardiovascular populations, however, both components are influenced by nutritional status, systemic inflammation, immune function, renal and hepatic function, fluid status, and overall disease severity [8,9,10]. PNI should therefore be interpreted as a composite immunonutritional/inflammatory marker rather than a nutrition-specific measure. PNI has been associated with outcomes in acute coronary syndrome, heart failure, and coronary artery disease populations undergoing PCI [11,12]. Evidence specifically in elderly (≥60 years) patients after successful CTO-PCI remains limited, motivating evaluation in this high-risk subgroup without implying that PNI itself is a novel biomarker.

Therefore, this study aims to systematically explore the association between PNI and long-term all-cause mortality and MACE after successful PCI in elderly CTO patients through a retrospective cohort, in order to provide more information for the clinical prognosis assessment of this population.

## 2. Data and Methods

### 2.1. Research Objects

**Figure 1:**
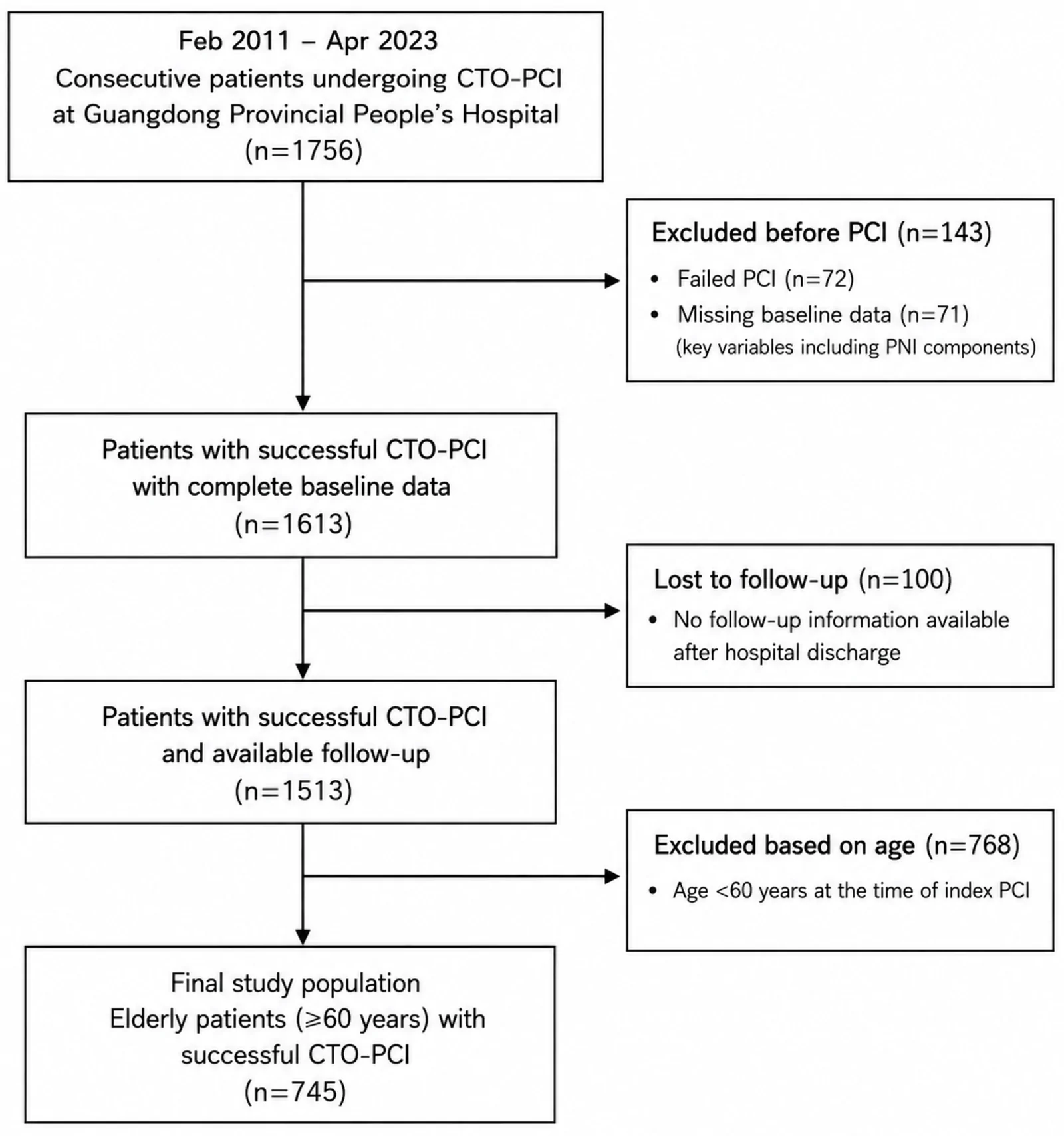
Flow diagram of sequential patient enrollment and exclusion in the CTO-PCI cohort.

This single-center retrospective cohort study initially screened 1756 consecutive patients who underwent PCI for CTO at Guangdong Provincial People’s Hospital between February 2011 and April 2023. We first excluded 72 patients with failed PCI and 71 with missing baseline data required for PNI calculation or prespecified covariates, leaving 1613 patients with successful CTO-PCI and complete baseline data. During follow-up, 100 patients without available follow-up information were excluded, leaving 1513 patients with successful CTO-PCI and available follow-up. To focus on the elderly population, 768 patients aged < 60 years were then excluded. The final analytic cohort therefore comprised 745 patients aged ≥ 60 years with successful CTO-PCI. Successful PCI was defined as restoration of TIMI grade 3 flow in the target CTO vessel without major in-hospital complications. The study protocol was approved by the Ethics Committee of Guangdong Provincial People’s Hospital and conducted in accordance with the Declaration of Helsinki. The patient-selection process is shown in Figure 1.

### 2.2. Assessment of PNI

PNI was calculated using the formula: PNI = serum albumin (g/L) + 5 × lymphocyte count (×10^9^/L), based on baseline laboratory values [7]. All included patients were divided into three groups according to their PNI tertiles: T1 group (low PNI, PNI ≤ 43.55, *n* = 249), T2 group (medium PNI, 43.55 < PNI ≤ 49.10, *n* = 248), and T3 group (high PNI, PNI > 49.10, *n* = 248).

### 2.3. Baseline Data Collection

Patient baseline data were systematically collected from the electronic medical records system. Basic data collection: (1) Patient’s basic clinical characteristics: collect the patient’s age, sex, admission blood pressure, heart rate, and smoking history. (2) The patient’s previous medical history: history of hypertension, diabetes, dyslipidemia, prior myocardial infarction (MI), PCI, coronary artery bypass grafting (CABG), and stroke. (3) Auxiliary examination: complete blood count (including white blood cells, neutrophils, lymphocytes, hemoglobin, and platelets), lipid profile, blood glucose, glycated hemoglobin, C-reactive protein (CRP), N-terminal pro-B-type natriuretic peptide (NT-proBNP), troponin T (TnT), organ function (liver function, renal function), left ventricular ejection fraction (LVEF), and coronary angiography data. (4) Medications on Admission: use of statins, β-blockers, dual antiplatelet therapy, angiotensin-converting enzyme inhibitors or angiotensin II receptor blockers, calcium channel blockers, and dapagliflozin.

### 2.4. Follow-Up and Endpoint Event Definitions

MACE was defined as a composite of all-cause mortality, cardiac death, non-fatal MI, stroke, and target-vessel revascularization. This definition was chosen to capture the full clinical burden of disease and to maintain consistency with major CTO-PCI registries, while allowing for component-level analyses to disentangle the drivers of any observed associations. All-cause mortality was selected as the primary endpoint because it is the most robust and objectively verifiable outcome, free from adjudication bias, and had the highest event count, providing adequate statistical power for multivariable adjustment. Cardiovascular death was adjudicated using predefined criteria (death from myocardial infarction, heart failure, arrhythmia, or sudden cardiac death, as documented by medical records and death certificates). Patients were followed through outpatient visits, telephone interviews, and medical-record review from the date of PCI until the first endpoint event, last available follow-up, or 25 June 2024, whichever occurred first. Median follow-up was 813 (IQR 390–1229) days.

### 2.5. Statistical Methods

Continuous variables are presented as mean ± standard deviation if normally distributed, and compared using one-way ANOVA; otherwise, they are presented as median (interquartile range) and compared using the Kruskal–Wallis test. Categorical variables are expressed as numbers (percentages) and compared using the chi-square or Fisher’s exact test, as appropriate.

Survival curves were estimated using the Kaplan–Meier method and compared with the log-rank test. Associations of baseline PNI with all-cause mortality and MACE were evaluated using Cox proportional hazards regression. PNI was modeled both continuously (per 1-unit increase) and categorically according to tertiles (T1, T2, and T3). Three sequential Cox proportional hazards models were constructed. Model 1 was unadjusted. Model 2, designated as the primary multivariable model, adjusted for age, sex, diabetes, hypertension, prior myocardial infarction, and smoking. This model was selected to balance control of clinically important confounders against the limited number of outcome events. Model 3 additionally adjusted for estimated glomerular filtration rate (eGFR), left ventricular ejection fraction (LVEF), C-reactive protein (CRP), multi-vessel disease, low-density lipoprotein cholesterol (LDL-C), uric acid, and NT-proBNP. Given the number of covariates relative to the number of events, Model 3 was considered exploratory and was used only to assess the robustness of the findings under more extensive adjustment. Accordingly, the primary interpretation and conclusions were based on Model 2. Hazard ratios (HRs) with 95% confidence intervals (CIs) are reported. Tests for linear trend across PNI tertiles were performed by entering the ordinal tertile variable as a continuous term.

Potential nonlinear dose–response relationships between PNI and each endpoint were examined in the primary analysis using restricted cubic spline (RCS) functions within the fully adjusted Cox model, with four knots placed at the 5th, 35th, 65th, and 95th percentiles of the PNI distribution. Overall association and nonlinearity were assessed using Wald tests. Prespecified subgroup analyses were performed according to sex, LVEF (≤40% vs. >40%), hypertension, diabetes, prior PCI, prior MI, eGFR (≤60 vs. >60 mL/min/1.73 m^2^), smoking, and dyslipidemia; effect modification was evaluated by adding multiplicative interaction terms to the Cox models.

Three sets of sensitivity analyses were prespecified to evaluate the robustness of the main findings. First, to reduce the risk of overfitting given the limited number of outcome events, an additional reduced-covariate Cox model was fitted. This model included PNI as the exposure of interest and age, NT-proBNP, uric acid, eGFR, LVEF, and CRP, which were associated with all-cause mortality at *p* < 0.05 in the univariable analyses. Although fewer covariates were included than in Model 3, the limited number of outcome events meant that the possibility of model overfitting could not be completely excluded. Accordingly, this analysis was considered exploratory and was used only to assess whether the direction and magnitude of the association remained consistent across alternative covariate specifications. Second, to assess possible calendar-period effects over the long recruitment interval, analyses were repeated after stratification by enrollment period (2011–2020 vs. 2021–2023), using the same PNI parameterization. Third, the robustness of the RCS findings to spline specification was evaluated using an alternative three-knot model with knots at the 10th, 50th, and 90th percentiles. These sensitivity analyses were intended to assess robustness rather than to define clinical thresholds.

All analyses were performed using R software (version 4.3.0). A two-sided *p* value < 0.05 was considered statistically significant.

## 3. Results

### 3.1. Baseline Characteristics of Patients

A total of 745 elderly CTO patients were included, with an overall median PNI of 46.15 (IQR: 43.55–49.10). As shown in Table 1, significant differences in baseline characteristics were observed across the three PNI tertiles. Compared with the T1 (lowest PNI) group, patients in the T3 (highest PNI) group were younger and exhibited more favorable cardiometabolic and inflammatory profiles. Specifically, the T3 group had significantly better renal function (eGFR), cardiac function (LVEF), and lipid profile (HDL-C), along with higher levels of hemoglobin and key blood cell counts. In contrast, markers of inflammation (CRP, fibrinogen, LDH, D-dimer), cardiac stress (NT-proBNP, TnT), and lipoprotein(a) were significantly lower in the T3 group (all *p* < 0.05). No significant differences were observed in most comorbidities, medications, or target vessel distribution, except for a lower prevalence of smoking history and prior MI in the T3 group.

Table S1 compares baseline characteristics between the 745 patients aged ≥ 60 years and the 768 patients aged < 60 years among patients with successful CTO-PCI and available follow-up. The ≥60-year cohort demonstrated significantly impaired renal function (eGFR: 76.4 vs. 94.2 mL/min/1.73 m^2^, *p* < 0.001) and left ventricular ejection fraction (56.0% vs. 59.0%, *p* = 0.028), alongside elevated neurohormonal stress (NT-proBNP: 389.4 vs. 139.7 pg/mL, *p* < 0.001) and myocardial injury markers (TnT: 21.89 vs. 14.35 ng/L, *p* < 0.001). Nutritional indices were notably compromised, evidenced by lower serum albumin (37.8 vs. 39.8 g/L), hemoglobin (131.0 vs. 140.0 g/L), and lymphocyte counts (1.67 vs. 1.92 × 10^9^/L), with concomitant elevation in systemic inflammation (CRP: 2.49 vs. 1.59 mg/L) (all *p* < 0.001). During a comparable follow-up duration (~2.2 years), elderly patients experienced significantly higher all-cause mortality (7.8% vs. 3.3%, *p* < 0.001) and cardiovascular mortality (4.8% vs. 2.2%, *p* = 0.006), with a trend toward increased MACE (13.6% vs. 10.5%, *p* = 0.072). Post-procedural β-blocker utilization was lower in the elderly group (76.4% vs. 83.6%, *p* < 0.001).

### 3.2. Association Analysis Between PNI and Prognosis

#### 3.2.1. Comparison of the Incidence of Endpoint Events

During the follow-up period, a total of 101 cases (13.6%) of MACEs and 58 cases (7.8%) of all-cause mortality occurred. The incidence of endpoint events differed significantly among tertiles. With increasing PNI levels, the incidence of all-cause mortality was significantly reduced (16.5% vs. 3.6% vs. 3.2%, *p* < 0.001); MACEs were significantly reduced (22.9% vs. 8.9% vs. 8.9%, *p* < 0.001) (Table 1).

#### 3.2.2. Kaplan–Meier Survival Analysis

**Figure 2:**
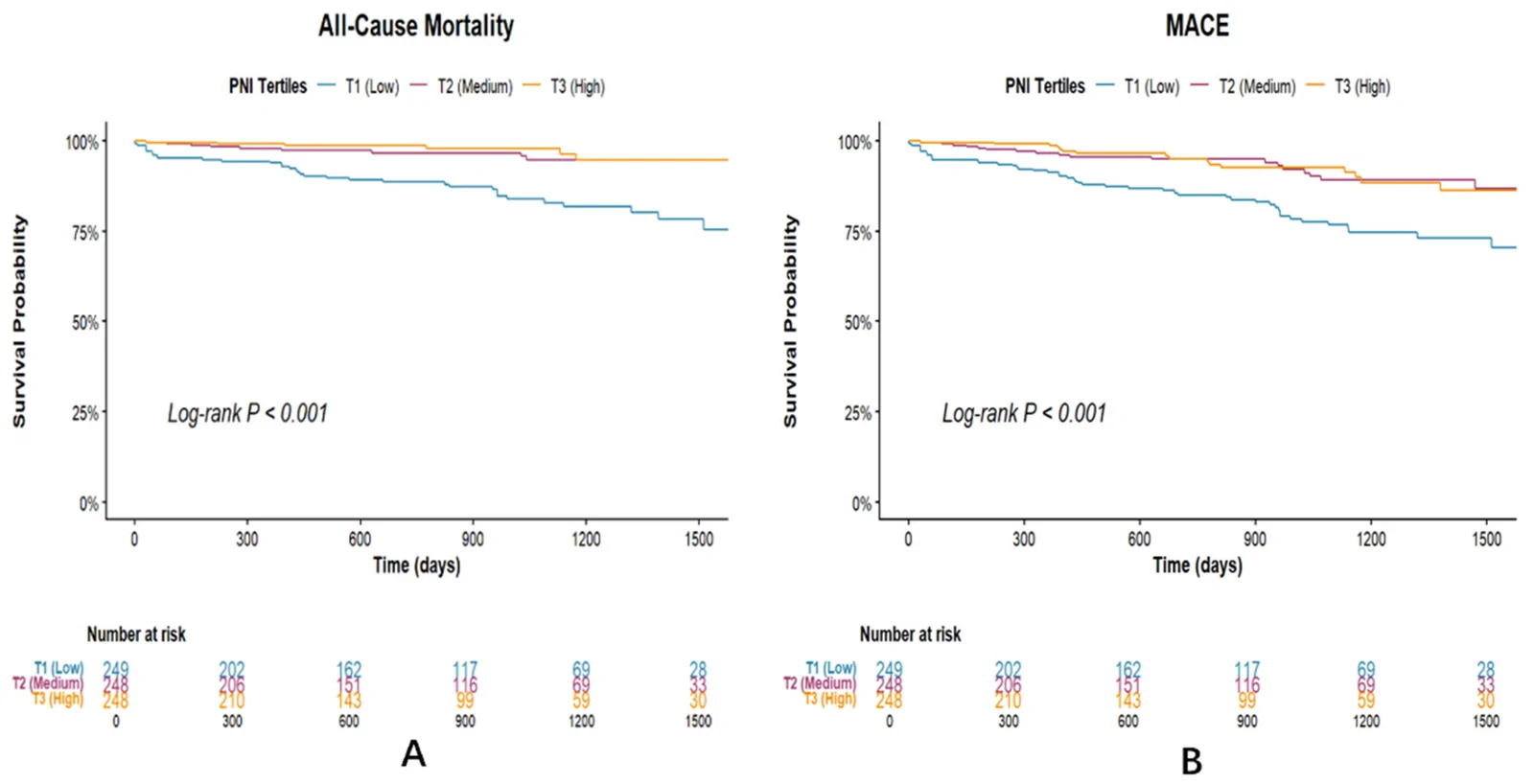
Kaplan–Meier survival curves for (**A**) all-cause death and (**B**) major adverse cardiovascular events. *p* values were obtained from log-rank tests comparing the three PNI-tertile curves.

Kaplan–Meier survival curves demonstrated a strong association between PNI levels and clinical outcomes (Figure 2). As shown in Figure 2A for all-cause mortality, the survival curves for the three tertiles diverged early during follow-up. The T1 group had the highest cumulative mortality, while mortality was significantly lower in the T2 and T3 groups (log-rank *p* < 0.001). Similarly, for MACE, event-free survival rates improved significantly with higher PNI levels. The incidence of MACE was markedly higher in the T1 group compared to the T2 and T3 groups (log-rank *p* < 0.001) (Figure 2B).

#### 3.2.3. Multivariate Cox Regression Analysis

In Cox regression analyses, higher PNI was associated with lower risks of both MACE and all-cause mortality (Table 2). In the unadjusted model (Model 1), each 1-unit increase in PNI was associated with lower risks of MACE (HR 0.91, 95% CI 0.87–0.94; *p* < 0.001) and all-cause mortality (HR 0.85, 95% CI 0.81–0.89; *p* < 0.001). In the primary multivariable model (Model 2), these associations remained significant for MACE (HR 0.92, 95% CI 0.88–0.96; *p* < 0.001) and all-cause mortality (HR 0.85, 95% CI 0.81–0.90; *p* < 0.001). When PNI was analyzed according to tertiles in Model 2, compared with the T1 group, the T2 group had a 62% lower risk of MACE (HR 0.38, 95% CI 0.23–0.63; *p* < 0.001) and a 79% lower risk of all-cause mortality (HR 0.21, 95% CI 0.10–0.44; *p* < 0.001). The T3 group had a 51% lower risk of MACE (HR 0.49, 95% CI 0.30–0.82; *p* = 0.007) and a 76% lower risk of all-cause mortality (HR 0.24, 95% CI 0.11–0.52; *p* < 0.001). Significant trends across increasing PNI tertiles were observed for both MACE (*p* for trend = 0.011) and all-cause mortality (*p* for trend < 0.001). The associations were directionally consistent in the more extensively adjusted Model 3 when PNI was analyzed as a continuous variable (MACE: HR 0.94, 95% CI 0.89–0.98; *p* = 0.006; all-cause mortality: HR 0.88, 95% CI 0.82–0.93; *p* < 0.001). However, given the limited number of outcome events relative to the number of included covariates, Model 3 was considered exploratory and was used only to assess the robustness of the primary Model 2 findings.

#### 3.2.4. Component-Level Analyses of MACE

Component-level analyses were performed to clarify the composite endpoint. There were 36 cardiovascular deaths, 35 TVR events, 5 non-fatal MIs, and 8 strokes. Because non-fatal MI and stroke were too infrequent for reliable multivariable modeling, adjusted analyses were limited to cardiovascular death and TVR. Higher PNI was associated with lower cardiovascular mortality (per 1-unit increase: HR 0.89, 95% CI 0.82–0.97; *p* = 0.005) but not with TVR (HR 1.01, 95% CI 0.92–1.10; *p* = 0.872) (Table S2). Thus, the MACE association appears to be driven predominantly by mortality rather than revascularization events and should not be interpreted as a uniform association across all MACE components.

### 3.3. Dose–Response Relationship Analysis

**Figure 3:**
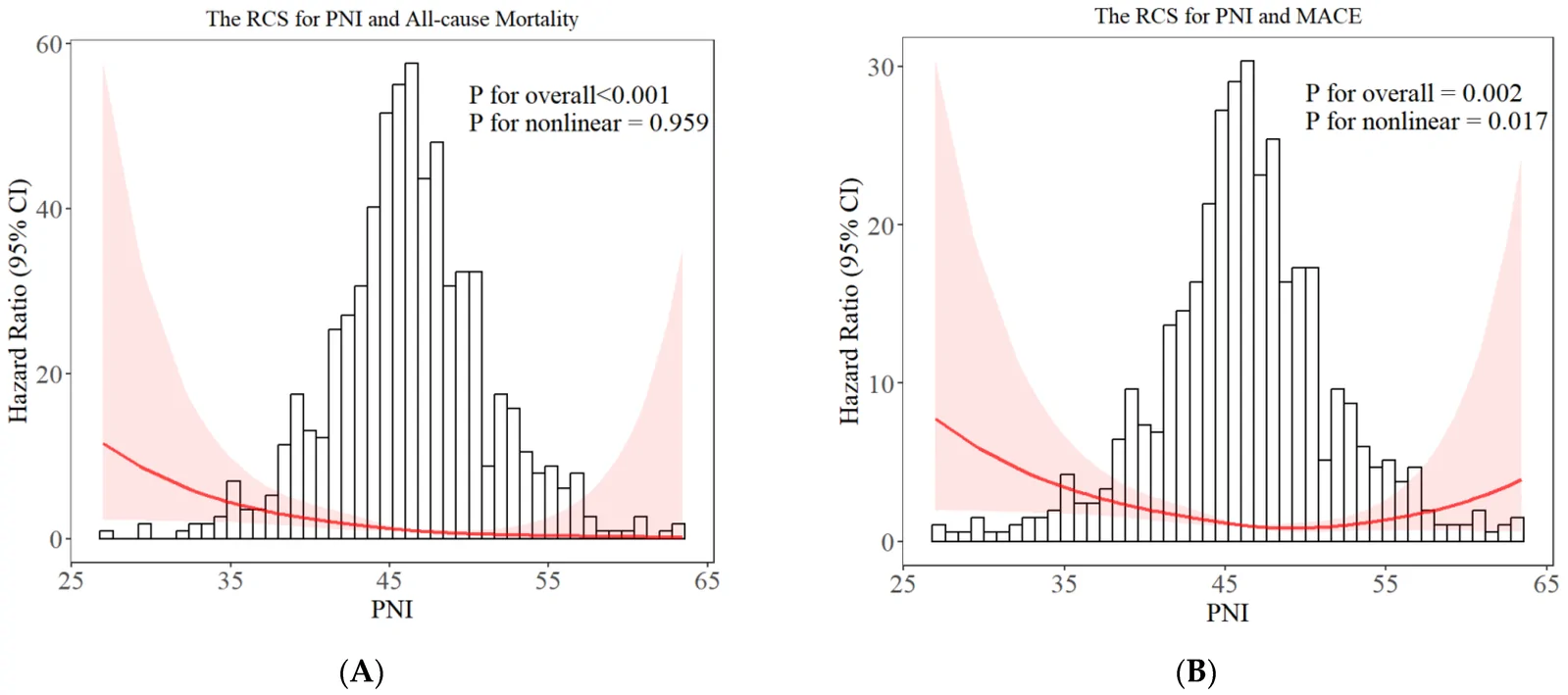
Restricted cubic spline analysis for (**A**) all-cause mortality and (**B**) major adverse cardiovascular events. *p* values are from Wald tests for the overall association and for nonlinearity of PNI in the adjusted Cox models.

Restriction cubic spline analysis revealed different dose–response patterns between PNI and endpoint events (Figure 3). For all-cause mortality, PNI showed a linear negative correlation with mortality risk (nonlinear *p* = 0.959), and the risk of death continued to flatten as PNI increased. The inflection point for this association was at a PNI of 46.3. In contrast, the association between PNI and MACE was nonlinear (*p* for nonlinearity = 0.017), characterized by two inflection points at PNI values of approximately 41.9 and 56.7. However, these data-derived turning points were considered exploratory rather than clinical cut-offs, particularly because observations were sparse in the upper tail of the PNI distribution.

### 3.4. Subgroup Analysis

**Figure 4:**
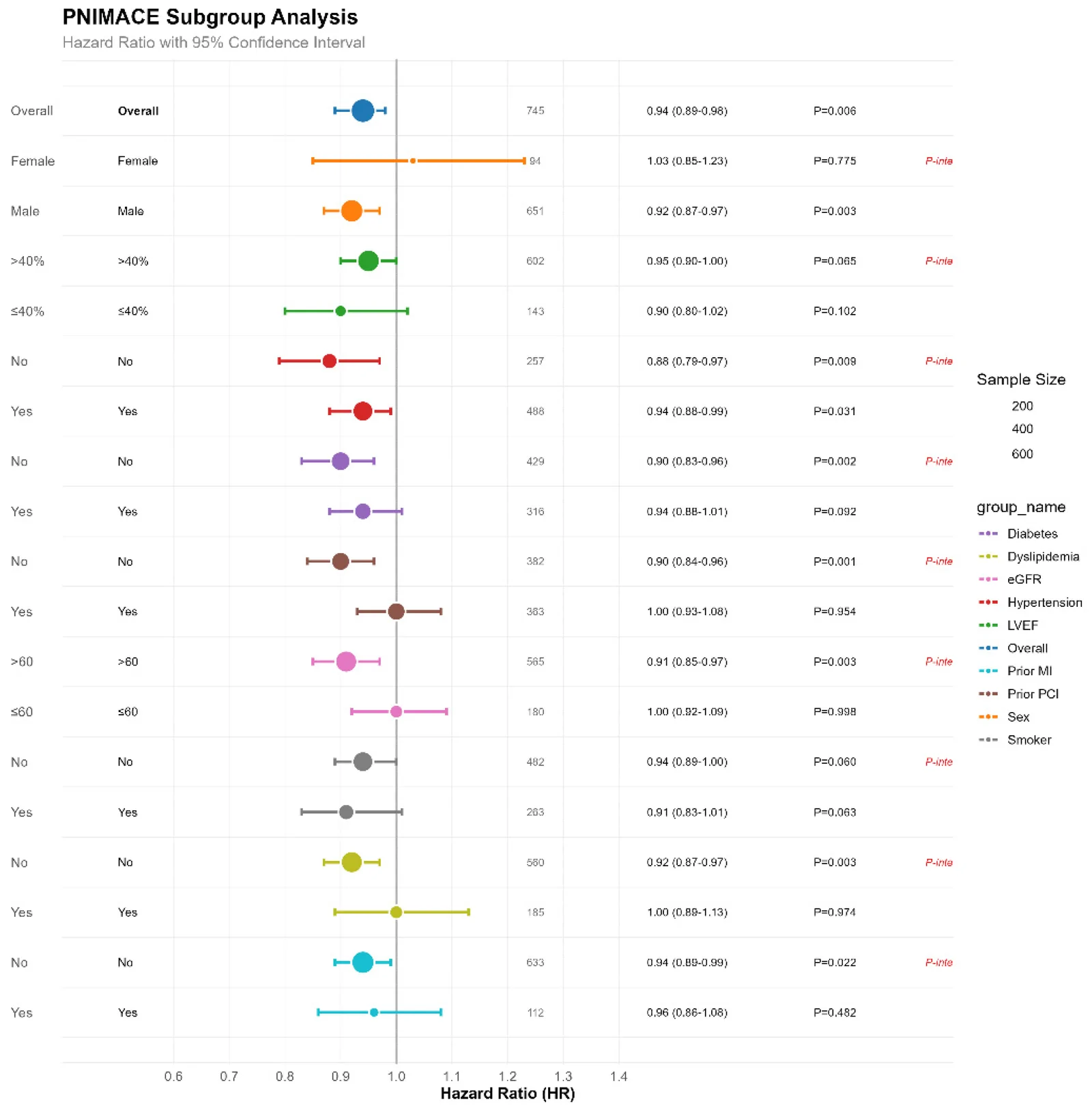
Subgroup analysis for all-cause mortality.

**Figure 5:**
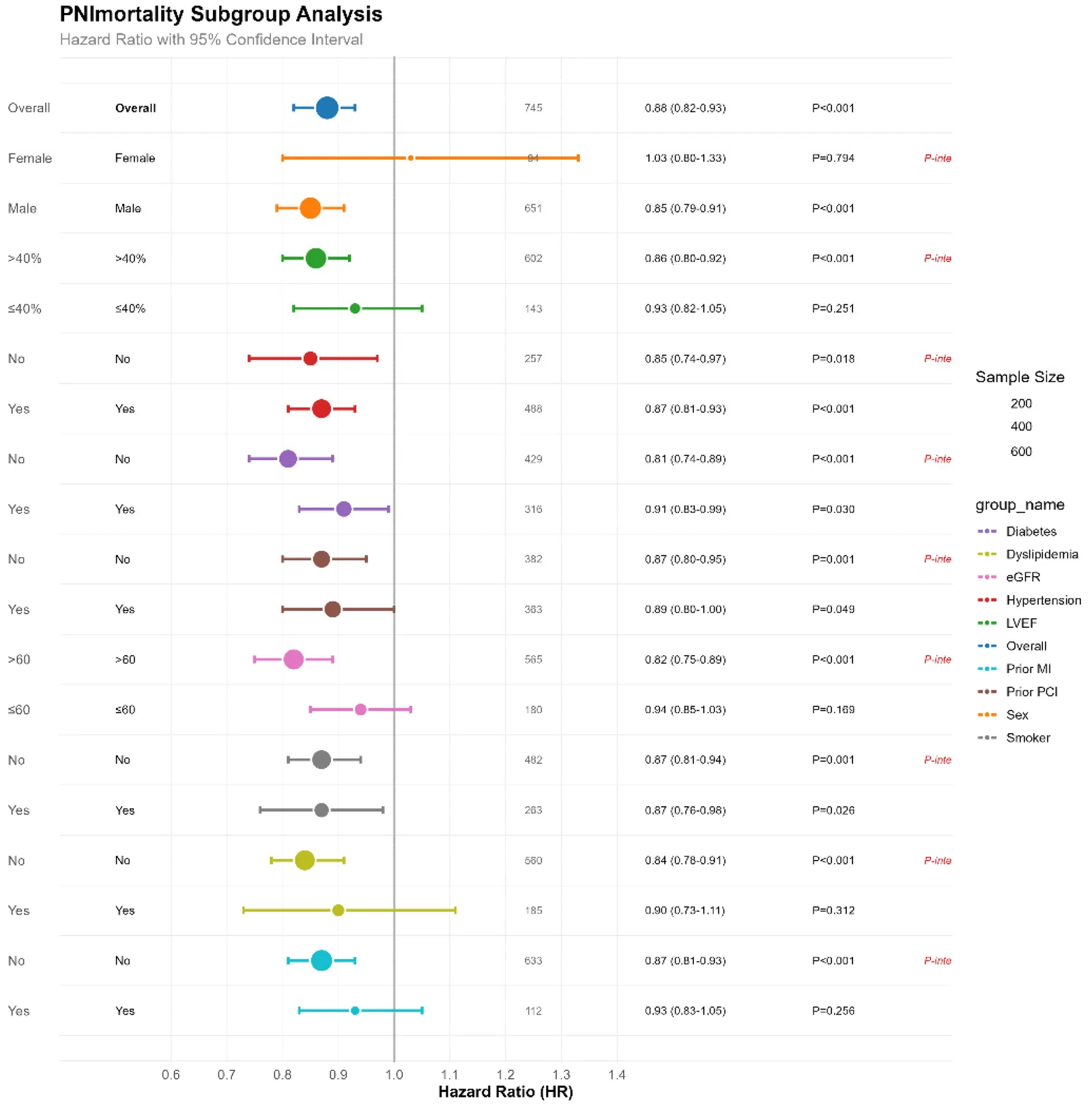
Subgroup analysis for major adverse cardiovascular events.

The results of the prespecified subgroup analyses are presented in Figure 4 and Figure 5. The protective association between higher PNI and lower risk of all-cause mortality was consistent across all examined subgroups, including those stratified by gender, LVEF level (≤40% vs. >40%), presence of hypertension, diabetes, or dyslipidemia, renal function (eGFR ≤ 60 vs. >60 mL/min/1.73 m^2^), smoking status, history of PCI, and history of MI. Formal tests for interaction were not significant for any subgroup variable (all *p* > 0.05), indicating that the association was robust across these different clinical characteristics. Similarly, the inverse association between PNI and MACE risk was consistent across all subgroups, with no significant interactions observed.

### 3.5. Sensitivity Analyses

#### 3.5.1. Parsimonious Cox Model

In the reduced-covariate exploratory model including age, NT-proBNP, uric acid, eGFR, LVEF, and CRP, each 1-unit increase in PNI remained associated with lower risks of all-cause mortality (HR 0.89, 95% CI 0.84–0.94; *p* = 0.013) and MACE (HR 0.94, 95% CI 0.90–0.98; *p* = 0.006) (Tables S3 and S4). These findings were directionally consistent with the primary Model 2 results. Nevertheless, because the number of outcome events remained limited relative to the number of model parameters, this analysis did not completely exclude model overfitting and should be interpreted as exploratory.

#### 3.5.2. Sensitivity Analysis Stratified by Enrollment Period

When analyses were stratified by enrollment period, the inverse association between PNI and all-cause mortality was observed in both periods (2011–2020: HR 0.89, 95% CI 0.82–0.97; *p* = 0.011; 2021–2023: HR 0.84, 95% CI 0.75–0.94; *p* = 0.002). For MACE, the association was not statistically significant in 2011–2020 (HR 0.96, 95% CI 0.90–1.02; *p* = 0.192) but was statistically significant in 2021–2023 (HR 0.88, 95% CI 0.80–0.97; *p* = 0.008) (Table S5). However, the difference in statistical significance between the two periods should not be interpreted as evidence of a temporal change in the association. Given the reduced sample size and number of events within each period, the period-specific estimates were less precise, and the observed difference in *p* values likely reflects limited statistical power.

#### 3.5.3. Sensitivity Analysis Using Alternative RCS Knot Specifications

RCS analyses were repeated using three knots at the 10th, 50th, and 90th percentiles (Figure S1). The alternative specification was consistent with the approximately linear inverse association between PNI and all-cause mortality and retained evidence of nonlinearity for MACE (*p* for nonlinearity < 0.05).

## 4. Discussion

This study evaluated the association between baseline PNI and long-term outcomes in 745 elderly patients after successful CTO-PCI. In the primary multivariable analysis based on Model 2, higher PNI was associated with lower risks of all-cause mortality and MACE. The direction of these associations remained consistent in exploratory models with more extensive covariate adjustment. The RCS analysis suggested an approximately linear inverse association with all-cause mortality and an exploratory nonlinear association with MACE. Component-level analyses further suggested that the association with MACE was driven predominantly by mortality rather than TVR. These findings support PNI as a potential integrated immunonutritional and inflammatory risk marker but do not establish causality or incremental predictive utility.

This study demonstrates that lower baseline PNI is independently associated with higher risks of all-cause mortality and MACE in elderly patients after successful CTO-PCI. This is highly consistent with previous research findings in the context of broader cardiovascular disease. For example, a large-scale cohort study of patients with stable coronary heart disease published by Wang et al. clearly showed that PNI is an independent predictor of all-cause mortality and MACE [13], which is completely consistent in direction and trend with the results we observed in the elderly CTO population, However, our subgroup analysis found that the relationship between PNI and death prognosis did not reach statistical significance, which may be due to population heterogeneity. This association is biologically plausible, as PNI—comprising albumin and lymphocyte count-captures the dual impact of nutritional status and systemic inflammation, both of which are key drivers of cardiovascular disease progression [14]. Many studies have also shown that decreased serum albumin levels and decreased blood lymphocyte counts are risk factors for the development of coronary atherosclerosis [15,16,17]. Liu et al. included 3561 Chinese patients with post-PCI CAD in a retrospective study, with an average follow-up time of about 37.59 ± 22.24 months, and their findings showed that PNI was an independent predictor in post-PCI CAD patients, with higher PNI indicating a lower incidence of adverse events [18]. Cheng et al.’s study also showed that PNI nutritional status in acute heart failure (AHF)-based patients was independently associated with short- and long-term total cardiovascular mortality [12]. As mentioned earlier, the value of PNI has been demonstrated, and our study fills a gap in nutritional risk studies specifically for elderly CTO patients after successful re-revascularization. Even after correction for a wide range of confounders, including renal function, cardiac function, inflammatory markers (e.g., CRP), and markers of myocardial stress (e.g., ProBNP), each unit increase in PNI was associated with an independent 12% (HR 0.88) reduction in all-cause death and a 6% independent reduction in MACE risk (HR 0.94). Aging predisposes elderly patients to hypoalbuminemia and lymphopenia due to impaired protein metabolism and thymic degeneration, directly lowering PNI levels [19,20,21]. Moreover, comorbidities common in elderly CTO patients, such as diabetes and renal insufficiency, promote chronic inflammation (e.g., TNF-α, IL-6), which suppresses albumin synthesis and exacerbates immune dysfunction, thereby further lowering PNI [22,23,24,25]. The innovative core finding of this study lies in the heterogeneous dose–response relationship between PNI and different endpoints. PNI is linearly negatively associated with all-cause mortality, consistent with Liu et al.’s study in patients with CAD [18]. However, the significant nonlinear relationship between PNI and MACE risk (nonlinear *p* = 0.017) is a more refined and novel observation. This is different from previous studies that suggested that higher PNI was associated with lower prognosis for patients with coronary artery disease after PCI [13,18,26].

The innovative core finding of this study lies in the heterogeneous dose–response relationship between PNI and different endpoints. PNI is linearly negatively associated with all-cause mortality, consistent with Liu et al.’s study in patients with coronary heart disease [13]. However, the significant nonlinear relationship between PNI and MACE risk (nonlinear *p* = 0.017) is a more refined and novel observation. This is different from previous studies that suggested that higher PNI was associated with lower prognosis for patients with coronary artery disease after PCI [13,14,15].

The clinical interpretation of the inflection points (PNI ≈ 41.9 and 56.7) is critical. They effectively stratify patients into three distinct risk-benefit categories: (1) a high-risk, high-intervention-benefit zone (PNI < 41.9), where patients face the highest MACE risk yet also experience the steepest decline in risk per unit increase in PNI; (2) a transitional-benefit zone (PNI 41.9–56.7); and (3) a lower-risk, diminishing-return plateau (PNI > 56.7), where further increases in PNI confer minimal additional reduction in MACE risk. The nonlinear association between PNI and MACE risk observed in our RCS analysis is an exploratory finding that requires independent validation. The identification of two inflection points (approximately PNI 41.9 and 56.7) suggests potential risk stratification thresholds; however, several caveats warrant emphasis. First, the upper inflection point is based on only 38 patients (5.1% of the cohort) with PNI > 54.84. Second, the apparent increase or plateau in MACE risk at very high PNI levels may represent a statistical artifact due to sparse data or residual confounding rather than a true biological phenomenon. Third, and most importantly, this observational association cannot establish causality, and we cannot infer that nutritional interventions targeting PNI elevation would modify clinical outcomes. Until validated in external cohorts and, ideally, in prospective interventional studies, these thresholds should be viewed solely as hypothesis-generating tools for future research, not as clinical targets. But a systematic review and meta-analysis on the Prognostic Nutritional Index and clinical outcomes in patients with acute MI did not reveal a significant association between PNI and the risk of MACE [27]. Therefore, this finding is an important difference from the simple linear relationship reported in most previous studies, and the plausible explanation lies in the intrinsic heterogeneity of MACE composite endpoints. The association between PNI and MACE reflects the diverse pathophysiology of its individual components—including death, MI, stroke, and TVR—each of which may be influenced by nutritional and inflammatory status to varying degrees. The observed MACE association is primarily driven by the mortality component, as PNI showed no significant association with TVR and the composite of non-fatal events. This pattern is biologically plausible, given that PNI reflects nutritional and inflammatory status, which are well-established determinants of mortality rather than restenosis or target-vessel revascularization. Notably, the median PNI level of the patients in this study (46.15) was higher than many of the norms reported in the heart failure or malignancy population, reflecting the difference between the relatively stable coronary heart disease patients and the end-stage disease patient population [7,12,28].

The enrollment-period sensitivity analysis showed an inverse association between PNI and all-cause mortality in both the 2011–2020 and 2021–2023 periods. For MACE, the association reached statistical significance only in the later period. However, statistical significance in one period but not in another does not establish that the association differed between periods. Because stratification substantially reduced the sample size and number of events available for each analysis, the difference in *p* values most likely reflects limited statistical power and imprecision rather than a genuine temporal change in the prognostic role of PNI. These period-specific findings should therefore be considered exploratory. Moreover, because the recruitment period spanned more than a decade, secular changes in PCI techniques, secondary prevention, and patient management may still have influenced the observed associations.

The strengths of this study include its focus on a specific high-risk elderly CTO-PCI population, relatively long follow-up, sequential multivariable adjustment, dose–response assessment using RCS, and multiple sensitivity and subgroup analyses. These analyses support the consistency of the observed associations but do not eliminate residual confounding inherent to the retrospective design. This study has several limitations. First, its single-center retrospective design is susceptible to selection bias and residual confounding. The cohort was restricted to patients aged ≥ 60 years with successful CTO-PCI, complete baseline data, and available follow-up; therefore, the findings should not be extrapolated to younger patients, patients with failed PCI, or patients not selected for intervention. The revised flow diagram makes each exclusion step explicit. Second, PNI was measured only once at baseline, and serial nutritional or inflammatory changes were unavailable. Detailed dietary intake, physical activity, and validated frailty assessments were not systematically collected; unmeasured frailty and global disease burden may therefore confound the observed associations. Third, the extended enrollment period (2011–2023) spanned substantial changes in PCI techniques, secondary-prevention strategies, and clinical management. Although the association between PNI and all-cause mortality was observed in both calendar periods, the period-stratified analyses had limited sample sizes and event numbers. Therefore, the difference in statistical significance for MACE across periods likely reflects limited power and imprecise estimates and should not be interpreted as evidence that the prognostic role of PNI changed over time. At the same time, residual confounding by unmeasured secular changes cannot be completely excluded. Fourth, the small numbers of non-fatal MI and stroke events precluded robust multivariable modeling of these components, and the TVR analysis remained limited in power. Fifth, the nonlinear RCS findings, especially the apparent upper turning point, were based on sparse observations and were sensitive to spline specification; these findings are hypothesis-generating rather than clinical thresholds. Sixth, the limited number of outcome events constrained the number of covariates that could be reliably included in the multivariable models. Although Model 2 was selected as the primary model and achieved an EPV of approximately 9.7, this value remains close to, rather than clearly above, the conventional threshold of 10 events per predictor. Moreover, the reduced-covariate sensitivity model still did not fully satisfy this conventional rule, and Model 3 included substantially more covariates relative to the number of events. Consequently, residual model instability and overfitting cannot be excluded. The Model 3 and reduced-covariate model results should therefore be regarded as exploratory, and the main conclusions are based on Model 2. Larger cohorts with more outcome events are needed to confirm the independence and stability of these associations. Finally, the study population was recruited in China, which may limit generalizability to other healthcare settings and populations. These observational data do not establish causality or demonstrate that interventions intended to increase PNI will improve clinical outcomes.

## 5. Conclusions

In this single-center retrospective cohort of elderly patients after successful CTO-PCI, lower baseline PNI was associated with higher risks of all-cause mortality and MACE. Prospective multicenter studies are required to validate the prognostic utility of PNI and to determine whether modification of nutritional or inflammatory status improves outcomes.

## Figures and Tables

**Table 1:** Baseline characteristics of the study population according to PNI tertiles.

Variable	All *N* = 745	T1 (Low) *N* = 249	T2 (Medium) *N* = 248	T3 (High) *N* = 248	*p*-Value
Age, years	67.00 (63.00, 71.00)	68.00 (65.00, 73.00)	67.00 (63.00, 71.00)	65.00 (63.00, 68.00)	<0.001
Male, n (%)	651 (87.4%)	225 (90.4%)	218 (87.9%)	208 (83.9%)	0.089
SBP, mmHg	133.00 (121.00, 147.00)	132.00 (118.00, 145.00)	133.00 (123.00, 148.00)	133.50 (123.00, 148.00)	0.039
DBP, mmHg	75.00 (68.00, 84.00)	73.00 (67.00, 81.00)	75.00 (67.00, 85.00)	79.00 (71.00, 84.00)	<0.001
HR, beats/min	73.00 (67.00, 81.00)	74.00 (67.00, 82.00)	72.00 (65.00, 79.50)	74.00 (68.00, 83.00)	0.046
HB, g/L	131.00 (120.00, 141.00)	124.00 (111.00, 136.00)	130.50 (121.50, 139.00)	135.00 (126.00, 145.00)	<0.001
WBC, 10^9^/L	6.82 (5.76, 8.09)	6.31 (5.38, 8.01)	6.73 (5.76, 7.64)	7.42 (6.36, 8.69)	<0.001
PLT, 10^9^/L	211.00 (174.00, 252.00)	202.00 (158.00, 242.00)	210.00 (174.50, 250.50)	218.50 (193.50, 259.50)	<0.001
NEUT(%)	0.62 (0.09)	0.66 (0.09)	0.61 (0.08)	0.58 (0.09)	<0.001
LYMPH, 10^9^/L	1.67 (1.30, 2.04)	1.29 (1.06, 1.58)	1.66 (1.42, 1.94)	2.10 (1.76, 2.65)	<0.001
Total bilirubin, μmol/L	11.50 (9.00, 14.50)	11.40 (8.60, 15.30)	11.20 (8.95, 14.15)	11.70 (9.45, 14.45)	0.4
CK, U/L	82.00 (60.00, 115.00)	80.00 (55.00, 120.00)	80.00 (60.00, 109.00)	86.50 (65.00, 117.00)	0.3
CK-MB, U/L	10.80 (10.00, 13.30)	10.70 (10.00, 13.50)	10.20 (10.00, 13.20)	11.00 (10.00, 13.60)	0.14
AST, U/L	22.00 (18.00, 27.00)	22.00 (18.00, 29.00)	21.00 (17.00, 26.50)	22.00 (18.00, 26.00)	0.4
ALT, U/L	20.00 (14.00, 29.00)	19.00 (13.00, 28.00)	20.00 (14.00, 30.00)	21.00 (15.00, 29.00)	0.077
LDH, U/L	166.00 (144.00, 194.00)	175.00 (149.00, 210.00)	164.50 (140.50, 188.00)	163.00 (145.00, 183.00)	<0.001
D-dimers, ng/mL	450.00 (300.00, 750.00)	560.00 (350.00, 1220.00)	420.00 (270.00, 700.00)	380.00 (280.00, 620.00)	<0.001
FIB, g/L	3.65 (3.17, 4.28)	3.77 (3.23, 4.61)	3.68 (3.12, 4.25)	3.53 (3.16, 4.05)	0.002
Lipoprotein a, mg/L	205.00 (79.00, 446.00)	328.00 (117.00, 596.00)	187.50 (71.50, 401.22)	167.50 (65.50, 341.50)	<0.001
Apolipoprotein A1, g/L	1.12 (1.01, 1.25)	1.09 (0.95, 1.19)	1.14 (1.03, 1.25)	1.15 (1.05, 1.28)	<0.001
LDL-C, mmol/L	2.33 (1.86, 2.88)	2.33 (1.77, 2.86)	2.31 (1.92, 2.83)	2.38 (1.90, 3.01)	0.6
HDL-C, mmol/L	0.93 (0.81, 1.08)	0.91 (0.78, 1.08)	0.93 (0.83, 1.09)	0.99 (0.86, 1.08)	0.006
TC, mmol/L	3.81 (3.20, 4.59)	3.66 (3.10, 4.61)	3.85 (3.20, 4.45)	3.95 (3.24, 4.73)	0.10
TG, mmol/L	1.38 (1.00, 1.90)	1.23 (0.90, 1.86)	1.33 (1.02, 1.84)	1.57 (1.09, 2.13)	<0.001
ALB, g/L	37.80 (35.70, 39.77)	35.26 (32.90, 36.76)	38.00 (36.47, 39.33)	39.90 (38.50, 42.49)	<0.001
UA, mmol/L	392.30 (321.60, 465.82)	385.50 (316.00, 472.48)	396.75 (330.30, 453.90)	392.98 (321.11, 468.70)	0.7
FBG, mmol/L	5.69 (4.93, 7.16)	5.87 (5.04, 7.85)	5.55 (4.89, 6.81)	5.66 (4.93, 6.96)	0.020
HbA1c, %	6.20 (5.80, 6.90)	6.20 (5.80, 6.90)	6.20 (5.80, 6.80)	6.20 (5.80, 7.10)	0.4
CRP, mg/L	2.49 (0.50, 7.10)	3.38 (0.80, 16.70)	2.44 (0.50, 5.84)	2.04 (0.50, 5.30)	<0.001
BUN, mmol/L	6.00 (4.80, 7.53)	5.99 (4.70, 8.49)	6.08 (5.02, 7.49)	5.87 (4.70, 6.79)	0.047
eGFR, ml/min/1.73 m^2^	76.35 (60.94, 89.62)	71.28 (55.81, 88.90)	78.08 (59.21, 89.35)	79.68 (66.58, 90.93)	0.001
ProBNP, pg/mL	389.40 (120.30, 1308.00)	845.60 (310.70, 2673.00)	258.25 (100.55, 956.50)	264.70 (93.64, 647.70)	<0.001
TNT, pg/mL	21.89 (11.80, 47.60)	29.50 (17.10, 85.80)	21.88 (10.80, 44.90)	16.40 (10.50, 28.50)	<0.001
LVEF, %	56.00 (44.00, 63.00)	54.00 (41.00, 62.00)	58.00 (45.00, 63.00)	60.00 (49.50, 64.00)	<0.001
**History**					
Hypertension, n (%)	488.0 (65.5%)	158.0 (63.5%)	169.0 (68.1%)	161.0 (64.9%)	0.5
Diabetes, n (%)	316.0 (42.4%)	103.0 (41.4%)	106.0 (42.7%)	107.0 (43.1%)	>0.9
Dyslipidemia, n (%)	185.0 (24.8%)	57.0 (22.9%)	67.0 (27.0%)	61.0 (24.6%)	0.6
Smoker, n (%)	263.0 (35.3%)	82.0 (32.9%)	77.0 (31.0%)	104.0 (41.9%)	0.025
Prior MI, n (%)	112.0 (15.0%)	43.0 (17.3%)	43.0 (17.3%)	26.0 (10.5%)	0.049
Prior CABG, n (%)	23.0 (3.1%)	6.0 (2.4%)	10.0 (4.0%)	7.0 (2.8%)	0.6
Stroke, n (%)	49.0 (6.6%)	16.0 (6.4%)	19.0 (7.7%)	14.0 (5.7%)	0.7
Prior PCI, n (%)	363.0 (48.7%)	116.0 (46.6%)	128.0 (51.6%)	119.0 (48.0%)	0.5
**Target CTO artery**					
RCA, n (%)	327.0 (43.9%)	107.0 (43.0%)	112.0 (45.2%)	108.0 (43.5%)	
RCA and LAD, n (%)	47.0 (6.3%)	14.0 (5.6%)	15.0 (6.0%)	18.0 (7.3%)	
RCA, LAD, and LCX, n (%)	12.0 (1.6%)	4.0 (1.6%)	2.0 (0.8%)	6.0 (2.4%)	
RCA and LCX, n (%)	23.0 (3.1%)	3.0 (1.2%)	14.0 (5.6%)	6.0 (2.4%)	
LAD, n (%)	273.0 (36.6%)	105.0 (42.2%)	85.0 (34.3%)	83.0 (33.5%)	
LAD and LCX, n (%)	33.0 (4.4%)	8.0 (3.2%)	11.0 (4.4%)	14.0 (5.6%)	
LCX, n (%)	30.0 (4.0%)	8.0 (3.2%)	9.0 (3.6%)	13.0 (5.2%)	
Multi-vessel disease, n (%)	673.0 (90.3%)	230.0 (92.4%)	221.0 (89.1%)	222.0 (89.5%)	0.4
Medicine					
Statin, n (%)	733.0 (98.4%)	242.0 (97.2%)	244.0 (98.4%)	247.0 (99.6%)	0.11
β-Blocker, n (%)	569.0 (76.4%)	191.0 (76.7%)	185.0 (74.6%)	193.0 (77.8%)	0.7
DAPT, n (%)	726.0 (97.4%)	244.0 (98.0%)	243.0 (98.0%)	239.0 (96.4%)	0.4
CCB, n (%)	169.0 (22.7%)	46.0 (18.5%)	55.0 (22.2%)	68.0 (27.4%)	0.057
ACEI, n (%)	436.0 (58.5%)	133.0 (53.4%)	153.0 (61.7%)	150.0 (60.5%)	0.13
DAPA, n (%)	138.0 (18.5%)	40.0 (16.1%)	47.0 (19.0%)	51.0 (20.6%)	0.4
**Followed-up**					
All-cause death, n (%)	58.0 (7.8%)	41.0 (16.5%)	9.0 (3.6%)	8.0 (3.2%)	<0.001
MACE, n (%)	101.0 (13.6%)	57.0 (22.9%)	22.0 (8.9%)	22.0 (8.9%)	<0.001
Cardiovascular mortality, n (%)	36.0 (4.8%)	23.0(9.2%)	8.0 (3.2%)	5.0 (2.0%)	<0.001
Non-fatal MI, n (%)	5.0 (0.7%)	3.0(1.2%)	2.0 (0.8%)	0.0 (0.0%)	0.4
Stroke, n (%)	8.0 (1.1%)	2.0(0.8%)	2.0 (0.8%)	4.0 (1.6%)	0.7
TVR, n (%)	35.0 (4.7%)	15.0 (6.0%)	10.0 (4.0%)	10.0 (4.0%)	0.5

Continuous variables are expressed as mean ± standard deviation or interquartile range, and categorical variables as frequencies (n) and percentages (%). Abbreviations: SBP, systolic blood pressure; DBP, diastolic blood pressure; HR, heart rate; RCA, right coronary artery; LAD, left anterior descending artery; LCX, left circumflex artery; LM, left main coronary artery; TC, total cholesterol; TG, triglyceride; LDL-C, low-density lipoprotein cholesterol; HDL-C, high-density lipoprotein cholesterol; WBC, white blood cell count; Hb, hemoglobin; PLT, platelet count; NEUT, neutrophils; LYMPH, lymphocytes; CK, creatine kinase; CK-MB, creatine kinase-MB; LDH, lactate dehydrogenase; FIB, fibrinogen; ALB, albumin; BUN, blood urea nitrogen; eGFR, estimated glomerular filtration rate; UA, uric acid; HbA1c, hemoglobin A1c; FBG, fasting blood glucose; CRP, C-reactive protein; LVEF, left ventricular ejection fraction; DAPT, dual antiplatelet therapy; DAPA, dapagliflozin; TVR, target-vessel revascularization; MI, myocardial infarction; MACE, major adverse cardiovascular events.

**Table 2:** Associations between PNI and MACE and all-cause mortality in participants with CTO.

	Model 1		Model 2		Model 3	
	HR (95%CI)	*p*-Value	HR (95%CI)	*p*-Value	HR (95%CI)	*p*-Value
**MACE, n (%)**						
As a continuous variable	0.91 (0.87–0.94)	<0.0001	0.92 (0.88–0.96)	<0.0001	0.94 (0.89–0.98)	0.006
T1	Reference		Reference		Reference	
T2	0.37(0.23–0.61)	<0.001	0.38 (0.23–0.63)	0.0002	0.43 (0.25–0.72)	0.001
T3	0.41 (0.25–0.67)	<0.001	0.49 (0.30–0.82)	0.007	0.60 (0.35–1.04)	0.072
*p* for trend		<0.001		0.011		0.0243
**All-cause mortality**						
As a continuous variable	0.85 (0.81–0.89)	<0.0001	0.85 (0.81–0.90)	<0.0001	0.88 (0.82–0.93)	<0.0001
T1	Reference		Reference		Reference	
T2	0.22 (0.10–0.44)	<0.001	0.21 (0.10–0.44)	<0.0001	0.28 (0.13–0.59)	0.0009
T3	0.20 (0.10–0.43)	<0.001	0.24 (0.11–0.52)	0.0003	0.36(0.16–0.81)	0.0142
*p* for trend		<0.001		<0.001		0.0017

Model 1 adjusted for: None. Model 2 adjusted for: age, sex, diabetes, hypertension, smoking, and Prior MI. Model 3 adjusted for: age, sex, diabetes, hypertension, smoking, Prior MI, multi-vessel disease, eGFR, CRP, LDL-C, UA, ProBNP, and LVEF.

## Data Availability

The original contributions presented in this study are included in the article/Supplementary Materials. Further inquiries can be directed to the corresponding author(s).

## Supplementary Materials

The following supporting information can be downloaded at https://www.mdpi.com/article/10.3390/jcdd13090464/s1, Table S1: Baseline characteristics according to age group among patients with successful CTO-PCI and available follow-up (<60 vs. ≥60 years); Table S2: Associations between PNI and clinical outcomes after successful CTO-PCI (*N* = 745); Table S3: Univariable Cox regression analysis for all-cause mortality; Table S4: Associations between PNI and clinical outcomes after successful CTO-PCI (adjusted for parsimonious model covariates) (*N* = 745); Table S5: Sensitivity analysis of the association between PNI and clinical outcomes stratified by enrollment period; Figure S1: Restricted cubic spline analysis of the association between PNI and (A) all-cause mortality and (B) major adverse cardiovascular events, with knots placed at the 10th, 50th, and 90th percentiles.
