# Supplementary material for: Association Between Prognostic Nutritional Index and Clinical Outcomes After Percutaneous Coronary Intervention in Elderly Patients with Chronic Total Occlusion
Source: J Cardiovasc Dev Dis. 2026 Sep 14;13(9):464. doi: 10.3390/jcdd13090464 (PMC13607444; doi:10.3390/jcdd13090464)
Supplement: Supplementary file 1 [file jcdd-13-00464-s001.zip › jcdd-4492928-supplementary.pdf]

Table S1. Baseline characteristics according to age group among patients with successful CTO-PCI and available follow-up (<60 vs. ≥60 years).

Variable	All N=1513	<60 years N=768	≥60 years N=745	*P*-value
Age, years	59.00 (52.00, 67.00)	52.00 (47.00, 56.00)	67.00 (63.00, 71.00)	<0.001
Male, n(%)	1,386.0 (91.6%)	735.0 (95.7%)	651.0 (87.4%)	<0.001
SBP, mmHg	130.00 (119.00, 143.00)	127.00 (116.00, 139.00)	133.00 (121.00, 147.00)	<0.001
DBP, mmHg	78.00 (70.00, 85.00)	79.00 (72.00, 87.00)	75.00 (68.00, 84.00)	<0.001
HR, beats/min	74.00 (67.00, 82.00)	75.00 (68.00, 83.50)	73.00 (67.00, 81.00)	0.064
eGFR, ml/min/1.73m^2	85.37 (68.03, 97.40)	94.18 (77.49, 103.38)	76.35 (60.94, 89.62)	<0.001
LVEF, %	58.00 (46.00, 63.00)	59.00 (48.00, 63.00)	56.00 (44.00, 63.00)	0.028
CRP, mg/L	1.97 (0.50, 6.20)	1.59 (0.50, 5.55)	2.49 (0.50, 7.10)	<0.001
LDL-C, mmol/L	2.39 (1.87, 2.95)	2.46 (1.90, 2.98)	2.33 (1.86, 2.88)	0.076
HDL-C, mmol/L	0.93 (0.82, 1.07)	0.92 (0.82, 1.05)	0.93 (0.81, 1.08)	0.047
TC, mmol/L	3.87 (3.20, 4.60)	3.90 (3.21, 4.64)	3.81 (3.20, 4.59)	0.12
TG, mmol/L	1.47 (1.06, 2.09)	1.57 (1.16, 2.34)	1.38 (1.00, 1.90)	<0.001
FBG, mmol/L	5.50 (4.85, 6.90)	5.30 (4.81, 6.70)	5.69 (4.93, 7.16)	<0.001
HbA1c, %	6.10 (5.70, 6.80)	6.00 (5.50, 6.60)	6.20 (5.80, 6.90)	<0.001
HB, g/L	135.00 (125.00, 146.00)	140.00 (130.00, 149.00)	131.00 (120.00, 141.00)	<0.001

Variable	All N=1513	<60 years N=768	≥60 years N=745	*P*-value
WBC, 10^9 /L	6.96 (5.84, 8.25)	7.07 (5.94, 8.32)	6.82 (5.76, 8.09)	0.024
PLT, 10^9/L	217.00 (180.00, 263.00)	224.00 (184.00, 267.50)	211.00 (174.00, 252.00)	<0.001
ALT, U/L	22.00 (16.00, 32.00)	24.29 (18.00, 33.00)	20.00 (14.00, 29.00)	<0.001
ProBNP, pg/ml	229.40 (73.40, 749.70)	139.70 (54.05, 454.50)	389.40 (120.30, 1,308.00)	<0.001
TNT, pg/ml	17.20 (9.70, 41.24)	14.35 (8.85, 33.24)	21.89 (11.80, 47.60)	<0.001
Lipoprotein a, mg/L	197.00 (75.00, 461.00)	187.50 (70.00, 491.00)	205.00 (79.00, 446.00)	0.3
BUN, mmol/L	5.59 (4.52, 6.83)	5.30 (4.40, 6.33)	6.00 (4.80, 7.53)	<0.001
Total bilirubin, μmol/L	11.70 (9.20, 14.90)	11.90 (9.45, 15.10)	11.50 (9.00, 14.50)	0.013
FIB, g/L	3.51 (3.07, 4.18)	3.38 (2.95, 4.01)	3.65 (3.17, 4.28)	<0.001
CK, U/L	87.00 (63.00, 122.00)	89.00 (66.00, 125.00)	82.00 (60.00, 115.00)	0.001
CK-MB, U/L	10.20 (10.00, 13.00)	10.00 (10.00, 12.60)	10.80 (10.00, 13.30)	<0.001
AST, U/L	22.00 (18.00, 27.00)	22.00 (18.00, 27.00)	22.00 (18.00, 27.00)	0.4
Apolipoprotein A1, g/L	1.11 (1.01, 1.24)	1.11 (1.01, 1.23)	1.12 (1.01, 1.25)	0.3
LDH, U/L	159.00 (139.00, 183.00)	152.00 (135.00, 179.00)	166.00 (144.00, 194.00)	<0.001
D-dimers, ng/ml	340.00 (240.00, 597.00)	270.00 (220.00, 430.00)	450.00 (300.00, 750.00)	<0.001
ALB, g/L	38.80 (36.56, 41.00)	39.80 (37.90, 41.70)	37.80 (35.70, 39.77)	<0.001

Variable	All N=1513	<60 years N=768	≥60 years N=745	*P*-value
NEUT(%)	0.60 (0.54, 0.66)	0.59 (0.54, 0.65)	0.61 (0.56, 0.68)	<0.001
LYMPH, 10^9/L	1.79 (1.43, 2.22)	1.92 (1.56, 2.39)	1.67 (1.30, 2.04)	<0.001
Hypertension, n (%)	902.0 (59.6%)	414.0 (53.9%)	488.0 (65.5%)	<0.001
Diabetes, n (%)	602.0 (39.8%)	286.0 (37.2%)	316.0 (42.4%)	0.040
Dyslipidemia, n (%)	423.0 (28.0%)	238.0 (31.0%)	185.0 (24.8%)	0.008
Smoker, n (%)	588.0 (38.9%)	325.0 (42.3%)	263.0 (35.3%)	0.005
Prior MI, n (%)	237.0 (15.7%)	125.0 (16.3%)	112.0 (15.0%)	0.5
Prior CABG, n (%)	36.0 (2.4%)	13.0 (1.7%)	23.0 (3.1%)	0.075
Stroke, n (%)	75.0 (5.0%)	26.0 (3.4%)	49.0 (6.6%)	0.004
Prior PCI, n (%)	757.0 (50.0%)	394.0 (51.3%)	363.0 (48.7%)	0.3
Target CTO artery				
RCA, n (%)	683.0 (45.1%)	356.0 (46.4%)	327.0 (43.9%)	
RCA and LAD, n (%)	98.0 (6.5%)	51.0 (6.6%)	47.0 (6.3%)	
RCA, LAD and LCX, n (%)	22.0 (1.5%)	10.0 (1.3%)	12.0 (1.6%)	
RCA and LCX, n (%)	55.0 (3.6%)	32.0 (4.2%)	23.0 (3.1%)	
LAD, n (%)	530.0 (35.0%)	257.0 (33.5%)	273.0 (36.6%)	

Variable	All N=1513	<60 years N=768	≥60 years N=745	*P*-value
LAD and LCX, (%)	63.0 (4.2%)	30.0 (3.9%)	33.0 (4.4%)	
LCX, n (%)	59.0 (3.9%)	29.0 (3.8%)	30.0 (4.0%)	
LM, n (%)	3.0 (0.2%)	3.0 (0.4%)	0.0 (0.0%)	
multi-vessel disease, n (%)	1,354.0 (89.5%)	681.0 (88.7%)	673.0 (90.3%)	0.3
Medicine				
Statin, n (%)	1,494.0 (98.7%)	761.0 (99.1%)	733.0 (98.4%)	0.2
β-Blocker, n (%)	1,211.0 (80.0%)	642.0 (83.6%)	569.0 (76.4%)	<0.001
DAPT, n (%)	1,477.0 (97.6%)	751.0 (97.8%)	726.0 (97.4%)	0.7
CCB, n (%)	318.0 (21.0%)	149.0 (19.4%)	169.0 (22.7%)	0.12
ACEI, n (%)	902.0 (59.7%)	466.0 (60.8%)	436.0 (58.5%)	0.4
Follow-up				
Time, days	810.00 (409.32, 1,230.00)	810.00 (425.00, 1,230.00)	813.00 (390.00, 1,228.93)	0.3
MACE, n (%)	182.0 (12.0%)	81.0 (10.5%)	101.0 (13.6%)	0.072
All-cause mortality, n (%)	83.0 (5.5%)	25.0 (3.3%)	58.0 (7.8%)	<0.001
Cardiovascular mortality, n (%)	53.0 (3.5%)	17.0 (2.2%)	36.0 (4.8%)	0.006
Non-fatal MI, n (%)	9.0 (0.6%)	4.0 (0.5%)	5.0 (0.7%)	0.7

Variable	All N=1513	<60 years N=768	≥60 years N=745	*P*-value
Stroke, n (%)	14.0 (0.9%)	6.0 (0.8%)	8.0 (1.1%)	0.6
TVR, n (%)	84.0 (5.6%)	49.0 (6.4%)	35.0 (4.7%)	0.2

Continuous variables are expressed as mean ± standard deviation or interquartile range, and categorical variables as frequencies (n) and percentages (%).

Abbreviations: SBP, systolic blood pressure; DBP, diastolic blood pressure; HR, heart rate; RCA, right coronary artery; LAD, left anterior descending artery; LCX, left circumflex artery; LM, left main coronary artery; TC, total cholesterol; TG, triglyceride; LDL-C, low-density lipoprotein cholesterol; HDL-C, high-density lipoprotein cholesterol; WBC, white blood cell count; Hb, hemoglobin; PLT, platelet count; NEUT, neutrophils; LYMPH, lymphocytes; CK, creatine kinase; CK-MB, creatine kinase-MB; LDH, lactate dehydrogenase; FIB, fibrinogen; ALB, albumin; BUN, blood urea nitrogen; eGFR, estimated glomerular filtration rate; UA, uric acid; HbA1c, hemoglobin A1c; FBG, fasting blood glucose; CRP, C-reactive protein; LVEF, left ventricular ejection fraction; DAPT, dual antiplatelet therapy; DAPA, dapagliflozin; TVR, target-vessel revascularization; MI, myocardial infarction; MACE, major adverse cardiovascular events.

Table S2. Associations between PNI and clinical outcomes after successful CTO-PCI (N = 745).

	Cardiovascular mortality		TVR	
	**HR (95%CI)**	***P*-value**	**HR (95%CI)**	***P*-value**
Per 1-unit increase in PNI	0.89 (0.82-0.97)	0.005	1.01 (0.92-1.10)	0.872
T1	Reference		Reference	
T2	0.33 (0.14-0.79)	0.013	0.52 (0.22-1.25)	0.144
T3	0.36 (0.13-1.02)	0.056	0.86 (0.35-2.13)	0.744

Models adjusted for age, sex, smoking, hypertension, diabetes, prior MI, multi-vessel disease, NT-proBNP, eGFR, LDL-C, UA, CRP, and LVEF.

HR, hazard ratio; CI, confidence interval; CTO, chronic total occlusion; PCI, percutaneous coronary intervention.

Table S3. Univariable Cox regression analysis for all-cause mortality.

**Variables**	**HR (95%CI)**	**P-value**
Age	1.55 (1.24 – 1.94)	< 0.001
NT-proBNP	1.54 (1.37 – 1.72)	< 0.001
uric acid	1.63 (1.32 – 2.01)	< 0.001
eGFR	0.49 (0.40 – 0.60)	< 0.001

Variables	HR (95%CI)	P-value
LVEF	0.67 (0.53 – 0.85)	< 0.001
CRP	1.23 (1.03 – 1.47)	0.005

Table S4. Associations between PNI and clinical outcomes after successful CTO-PCI (adjusted for parsimonious model covariates) (N = 745).

	All-cause mortality		MACEs	
	**HR (95%CI)**	***P*-value**	**HR (95%CI)**	***P*-value**
Per 1-unit increase in PNI	0.89 (0.84-0.94)	0.013	0.94 (0.90-0.98)	0.006
T1	Reference		Reference	
T2	0.30 (0.14-0.63)	0.001	0.46 (0.28-0.77)	0.003
T3	0.35 (0.16-0.78)	0.010	0.58 (0.34-0.98)	0.045

Models adjust for: age, NT-proBNP, eGFR, UA, CRP, and LVEF.
HR, hazard ratio; CI, confidence interval; CTO, chronic total occlusion; PCI, percutaneous coronary intervention.

Table S5. Sensitivity analysis of the association between PNI and clinical outcomes stratified by enrollment period.

	All-cause mortality		MACEs	
	**HR (95%CI)**	***P*-value**	**HR (95%CI)**	***P*-value**
2011-2020 (N = 349)				
Per 1-unit increase in PNI	0.89 (0.82-0.97)	0.011	0.96 (0.90-1.02)	0.192
2021-2023 (N = 396)				
Per 1-unit increase in PNI	0.84 (0.75-0.94)	0.002	0.88 (0.80-0.97)	0.008

Models adjust for: age, sex, smoking, hypertension, diabetes, Prior MI, multi-vessel disease, NT-proBNP, eGFR, LDL-C, UA, CRP, and LVEF

HR, hazard ratio; CI, confidence interval; CTO, chronic total occlusion; PCI, percutaneous coronary intervention.

Figure S1. Restricted cubic spline analysis of the association between PNI and (A) all-cause mortality and (B) major adverse cardiovascular events, with knots placed at the 10th, 50th, and 90th percentiles.

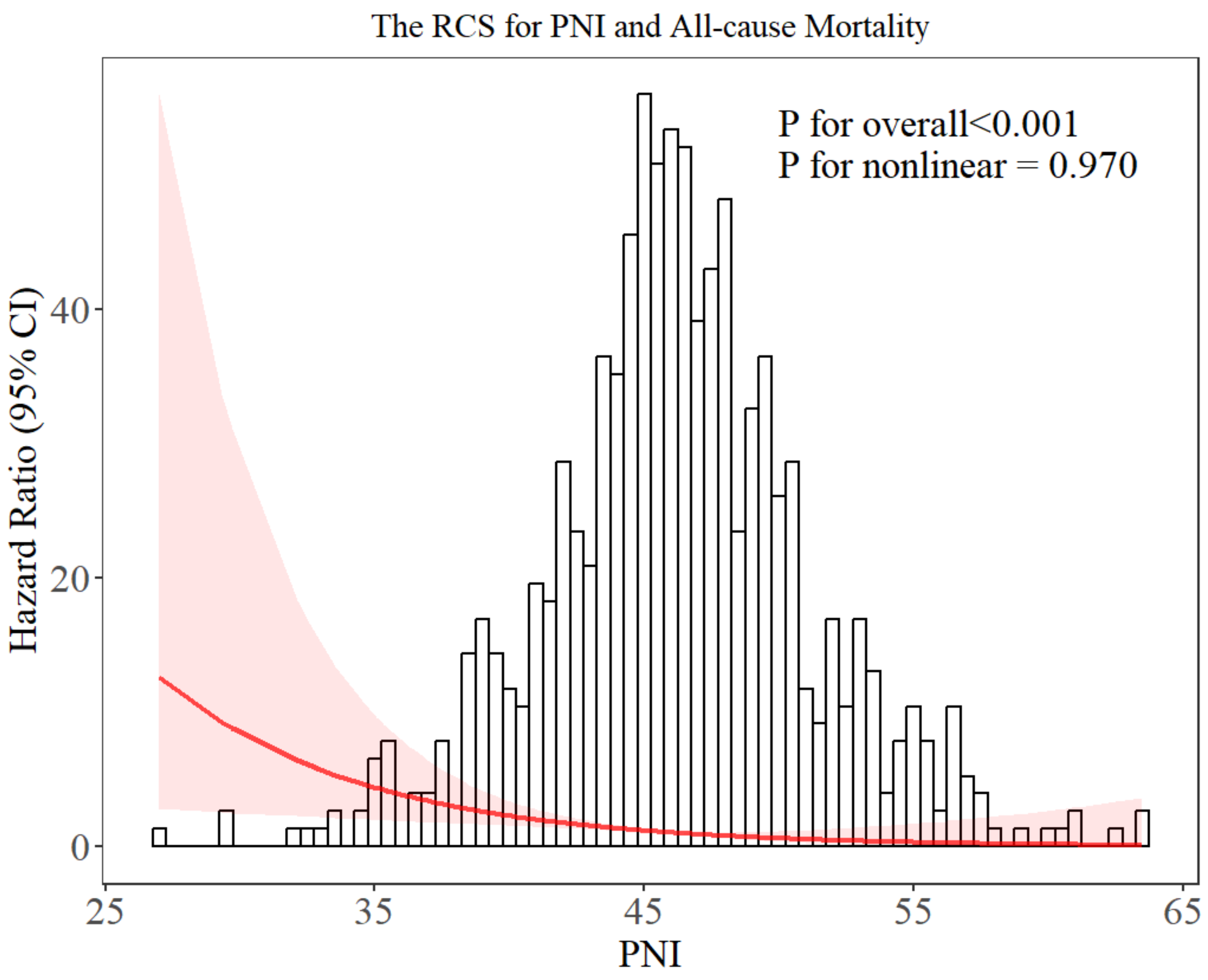
The RCS for PNI and All-cause Mortality
P for overall<0.001
P for nonlinear = 0.970
Hazard Ratio (95% CI)
40
20
0
25
35
45
55
65
PNI

A

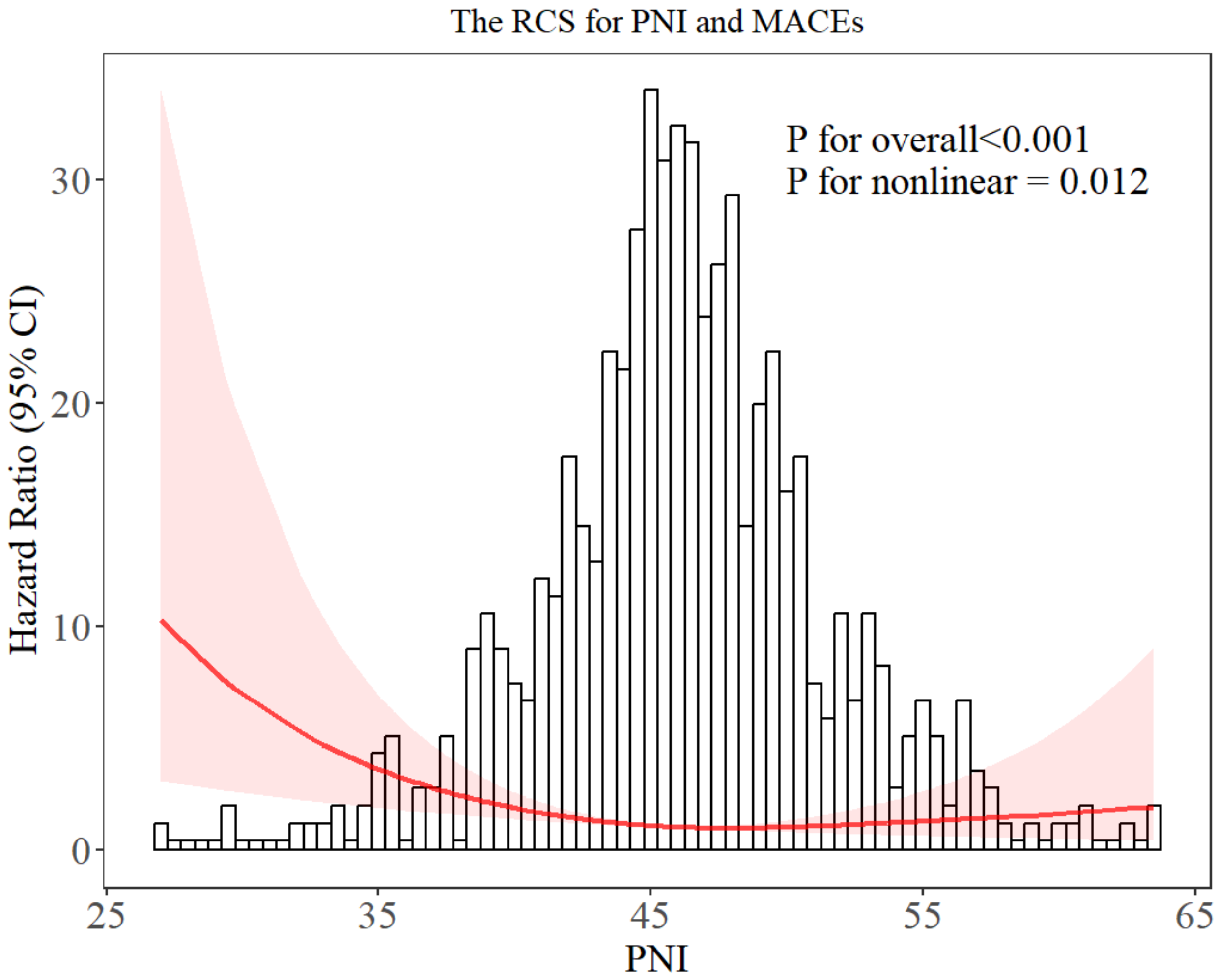
The RCS for PNI and MACEs
P for overall<0.001
P for nonlinear = 0.012
Hazard Ratio (95% CI)
30
20
10
0
25
35
45
55
65
PNI

B
